# IGF-1R Inhibitor Ameliorates Neuroinflammation in an Alzheimer’s Disease Transgenic Mouse Model

**DOI:** 10.3389/fncel.2020.00200

**Published:** 2020-07-03

**Authors:** Mona Sohrabi, Angela M. Floden, Gunjan D. Manocha, Marilyn G. Klug, Colin K. Combs

**Affiliations:** ^1^Department of Biomedical Sciences, University of North Dakota School of Medicine and Health Sciences, Grand Forks, ND, United States; ^2^Department of Geriatrics, University of North Dakota School of Medicine and Health Sciences, Grand Forks, ND, United States; ^3^Department of Population Health, University of North Dakota School of Medicine and Health Sciences, Grand Forks, ND, United States

**Keywords:** Alzheimer’s disease, neuroinflammation, Aβ, trophic factor, picropodophyllin

## Abstract

Aging is a major risk factor for Alzheimer’s disease (AD). Insulin-like growth factor-1 receptor (IGF-1R) regulates general aging and lifespan. However, the contribution of IGF-1 to age-related AD pathology and progression is highly controversial. Based on our previous work, AβPP/PS1 double transgenic mice, which express human mutant amyloid precursor protein (APP) and presenilin-1 (PS-1), demonstrated a decrease in brain IGF-1 levels when they were crossed with IGF-1 deficient Ames dwarf mice (df/df). Subsequently, a reduction in gliosis, amyloid-β (Aβ) plaque deposition, and Aβ_1–40/42_ concentrations were observed in this mouse model. This supported the hypothesis that IGF-1 may contribute to the progression of the disease. To assess the role of IGF-1 in AD, 9–10-month-old male littermate control wild type and AβPP/PS1 mice were randomly divided into two treatment groups including control vehicle (DMSO) and picropodophyllin (PPP), a selective, competitive, and reversible IGF-1R inhibitor. The brain penetrant inhibitor was given ip. at 1 mg/kg/day. Mice were sacrificed after 7 days of daily injection and the brains, spleens, and livers were collected to quantify histologic and biochemical changes. The PPP-treated AβPP/PS1 mice demonstrated attenuated insoluble Aβ_1–40/42_. Additionally, an attenuation in microgliosis and protein p-tyrosine levels was observed due to drug treatment in the hippocampus. Our data suggest IGF-1R signaling is associated with disease progression in this mouse model. More importantly, modulation of the brain IGF-1R signaling pathway, even at mid-life, was enough to attenuate aspects of the disease phenotype. This suggests that small molecule therapy targeting the IGF-1R pathway may be viable for late-stage disease treatment.

## Introduction

Alzheimer’s disease (AD), as the most common form of dementia, comprises 60–80% of all cases (Alzheimer’s Association, 2016). Extracellular amyloid β (Aβ) plaques and intracellular neurofibrillary tangles are AD pathological features hypothesized to lead to neuronal death and cognitive dysfunction (Terry, 1963; Glenner and Wong, 1984). Since aging is the main risk factor for AD, slowing down this process may delay disease onset or progression (Guerreiro and Bras, 2015). The growth hormone (GH)/insulin-like growth factor (IGF-1) signaling pathway is hypothesized to be one of the primary pathways regulating lifespan in general. Partial inactivation of the IGF-1 receptor (IGF-1R) gene or insulin-like signaling extends longevity and postpones age-related dysfunction in nematodes, flies, and rodents (Holzenberger et al., 2003; Tatar et al., 2003; Taguchi et al., 2007; Kappeler et al., 2008; Kenyon, 2010; Xu et al., 2014). Also, genetic mutations in the human IGF-1R, GH-R or low levels of IGF-1 correlate with lifespan extension (Suh et al., 2008; Guevara-Aguirre et al., 2011; Milman et al., 2014; van der Spoel et al., 2015). Therefore, pharmacologic inhibition of the GH/IGF-1 pathway is a promising strategy for impeding or slowing down age-related diseases (Longo et al., 2015).

However, the role of IGF-1 in regulating age-associated AD remains unclear. For instance, lower serum IGF-1 levels correlate with increased cognitive decline and risk of AD (Okereke et al., 2007; Westwood et al., 2014). Also, patients with familial AD demonstrate lower levels of circulating IGF-1 compared to controls (Mustafa et al., 1999). An *ex vivo* study revealed IGF-1 resistance along with insulin resistance through the PI3K pathway in AD patient brains (Talbot et al., 2012). Finally, IGF-1 treatment diminished Aβ accumulation by improving its transportation out of the brains of AD mouse models while IGF-1R inhibition aggravated both behavioral and pathological AD symptoms in mice (Carro et al., 2002, 2006).

On the other hand, the administration of a potent inducer of circulating IGF-1 levels (MK-677) failed to delay AD progression in a randomized trial (Sevigny et al., 2008). Also, acute or chronic delivery of IGF-1 exerted no beneficial effect on AD pathological hallmarks in rodent models *in vivo* (Lanz et al., 2008). Moreover, high levels of serum IGF-1 were detected in individuals diagnosed with AD or other forms of dementia in one cross-sectional study (Johansson et al., 2013). Decreased insulin/IGF-1 signaling (IIS) in aged women carrying polymorphisms which lead to reduced IIS activity correlated with better cognitive behavior in another study (Euser et al., 2008). In a familial study, higher serum IGF-1 at midlife increased the risk of late-onset AD (van Exel et al., 2014). Middle-aged and older males with high circulating IGF-1 levels at baseline were diagnosed with cognitive impairment after approximately 8 years in a longitudinal study (Tumati et al., 2016). Higher prevalence and incidence of dementia and AD were associated with higher levels of IGF-1R stimulating activity in an elderly, population-based cohort study (de Bruijn et al., 2014). Consistent with these findings, a body of studies have demonstrated that genetically ablating IGF-1R signaling improves neuroprotection and protects against AD progression by alleviating AD hallmarks including Aβ deposition, neuroinflammation, neuronal and synaptic loss, and behavioral dysfunction in AD mouse models (Cohen et al., 2009; Freude et al., 2009; Gontier et al., 2015; George et al., 2017).

Presumably, this dichotomy of effects is, in part, mediated through the effects of IGF-1 on its receptor. The IGF-1R and the insulin receptor (IR) are homologous tyrosine kinase proteins with remarkably different functions (Rothenberg et al., 1990; Larsson et al., 2005; Arcaro, 2013). Upon binding of IGF-1R ligands (IGF-1, IGF-2, and supraphysiological concentration of insulin), the receptor becomes auto-phosphorylated on three key tyrosine residues (Y1131, Y1135, and Y1136) in the activation loop (LeRoith et al., 1995; Baserga, 1999; Favelyukis et al., 2001). Phosphorylation of this receptor leads to activation of two major signaling pathways including RAS/RAF/MEK/ERK and PI3K/Akt which results in proliferation and protein synthesis/anti-apoptosis/autophagy, respectively (Gallagher and LeRoith, 2010; Gontier et al., 2015). Inhibition of IGF-1R-mediated signaling is considered a viable therapeutic strategy against cancer, including glioblastoma, to confront tumor growth (Girnita et al., 2004; Yin et al., 2010). Picropodophyllin (PPP), a cyclolignan compound, is a potent, selective, competitive, and reversible inhibitor that targets IGF-1R autophosphorylation at the substrate level (Girnita et al., 2004). Thus, it has no described effect on the function of IR or other tyrosine kinase receptors (Girnita et al., 2004; Vasilcanu et al., 2008). Inhibition of IGF-1R by PPP preferentially downregulates the PI3K/Akt signaling pathway through blocking activation loop phosphorylation (Vasilcanu et al., 2004).

Based on the potential role of IGF-1R suppression against AD development and according to our previous data which reported diminished gliosis and Aβ burden in the df/df/AβPP/PS1 transgenic mice which express low levels of brain IGF-1 (Puig et al., 2016), we hypothesized that applying a short-term pharmaceutical intervention might attenuate disease presentation. To test this idea, we intraperitoneally injected PPP into the AβPP/PS1 mouse line and wild type littermate controls for a week to investigate changes in gliosis and plaque deposition.

## Materials and Methods

### Animals

In this study, the wild-type (WT) C57BL/6 mouse line and the C57BL6 AβPP/PS1 (strain 005864 B6.Cg-Tg (AβPPswe, PSEN1dE9)85Dbo) transgenic mice were originally purchased from the Jackson Laboratory (Bar Harbor, ME, USA) and maintained, as a colony, under standard housing conditions including a 12 h light:12 h dark cycle and 22 ± 1°C temperature with *ad libitum* access to food and water at the University of North Dakota Center for Biomedical Research. This transgenic mouse model of AD expresses the human amyloid beta (A4) precursor protein (hAβPP) and the human presenilin 1 (hPSEN1), respectively, carrying the Swedish and deltaE9 mutations under the control of the mouse prion promoter. AβPP/PS1 mice develop significant amyloid plaque deposition and gliosis in their brains by 6–7 months of age. Due to limited animal availability, only male littermate control WT and AβPP/PS1 mice at 9–10 months of age (*n* = 7–9 per genotype) were used. They were randomly divided into the vehicle and drug-treated groups for 7-day treatments. Twenty-four hours (day 8) after ending the treatments, mice were euthanized followed by cardiac perfusion, and the blood, brains, spleens, and livers were collected to quantify histologic and biochemical changes. All procedures involving animals were reviewed and approved by the UND Institutional Animal Care and Use Committee (UND IACUC). The investigation conforms to the National Research Council of the National Academies Guide for the Care and Use of Laboratory Animals (8th edition).

### Adult Microglia Cultures

To assess an age-relevant microglial response to IGF-1 stimulation, adult microglia were isolated. Briefly, cortices were collected from 10-month-old male C57BL/6 mice in DMEM/F12 (Thermo Fisher Scientific, Waltham, MA, USA) + 0.1% BSA. Cortices were finely minced and digested with 3 μl each of 10 mg/ml papain and 5.5 mM L-cysteine-HCL in 3 ml/brain for 1 h @ 37°C on a rotator. Digested tissue was homogenized with a glass homogenizer 4–5X. Myelin was separated using a 30% Percoll gradient. The remaining cell suspension was filtered through 70 μm cell strainers. Cells were isolated further using Stemcell Technologies CD11b Positive selection kit II following the manufacturer’s protocol (Stemcell Technologies, Vancouver, Canada). Experiments were set up in 96-well plates with 100 μl/well of DMEM/F12 media without serum or antibiotics.

### Antibodies and Reagents

The Y188 antibody targeting AβPP was purchased from Abcam (Cambridge, MA, USA). The 4G10 antibody to target p-tyrosine (05–321) was purchased from Millipore (Darmstadt, Germany). The anti-oligomer antibody, A11, and anti-fibrillar protein antibody, OC, were gifts from Rakez Kayed. Anti-Aβ 6E10 antibody was purchased from Covance (Emeryville, CA, USA). Iba-1 and CD68 (MCA1957) antibodies against microglia markers were purchased from Wako Chemicals USA, Incorporation (Richmond, VA, USA) and BIO-RAD (CA, USA), respectively. Antibodies targeting α-tubulin (TU-02: sc-8035), actin (I-19: sc-1616), and GAPDH (6C5: sc-32233) were purchased from Santa Cruz Biotechnology (Santa Cruz, CA, USA). Antibodies against GFAP (D1F4Q) to target astrocytes, p-AKT (Ser473; 193H12), AKT (pan; C67E7), p-p44/42 MAPK (p-ERK1/2; Thr202/Tyr204), p44/42 MAPK (ERK1/2), IGF-1R β, p-SAPK/JNK (Thr183/Tyr185), and SAPK/JNK were purchased from Cell Signaling Technology, Incorporation (Danvers, MA, USA). To detect p-IR/IGF1-R (pYpY1162/1163) for western blotting antibodies were purchased from Abcam (Cambridge, MA, USA) and Life Technologies (Grand Island, NY, USA), respectively. The 4G8 antibody against the Aβ peptide was purchased from Biolegend (San Diego, CA, USA). The mouse on mouse (M.O.M) kit, Vector VIP kit, biotinylated anti-rat, anti-rabbit, and anti-mouse antibodies were purchased from Vector Laboratories Incorporation (Burlingame, CA, USA). The horseradish peroxidase-conjugated secondary antibodies were purchased from Santa Cruz Biotechnology (Santa Cruz, CA, USA). PPP, IGF-1R inhibitor, was purchased from EMD Millipore (Billerica, MA, USA). The cytokine ELISA kits were purchased from R&D Systems (Minneapolis, MN, USA). The human Aβ_1–40/42_ ELISA kits were purchased from EMD Millipore (Billerica, MA, USA).

### Intraperitoneal Injection of PPP

The male AβPP/PS1 and age-matched WT mice were randomly divided into two treatment (drug and vehicle) groups per genotype. PPP dissolved in DMSO was given to drug-treated groups with a dosage of 1 mg/kg/day while the vehicle groups received DMSO *via* ip. injection, totaling 7 days. A pilot assessment verifying drug concentration (1 mg/kg) efficacy for attenuating p-IGF-1R and p-tyrosine-protein levels in the hippocampus was performed by collecting brains 15 min after the PPP injection (Supplementary Figure S1).

### Tissue Enzyme-Linked Immunosorbent Assays (ELISA)

On the collection day, spleen and right temporal cortices of brain hemispheres were isolated and flash frozen. Temporal cortices were collected as a brain region of relevance in AD pathophysiology which also provided an appropriate amount of tissue for performing ELISA assays. Temporal cortices and spleens were lysed in Raybiotech lysis buffer and centrifuged (17,968 *g*, 4°C, 10 min) and cytokine and soluble Aβ_1–40/42_ ELISAs were performed from the supernatants according to the manufacturer’s protocol. The temporal cortices pellets were resuspended in 5 M guanidine HCL/50 mM Tris HCL, pH 8.0, centrifuged (17,968 *g*, 4°C, 10 min), and the supernatants were removed to quantify insoluble Aβ_1–40/42_ levels by ELISA according to the manufacturer’s protocol. The Bradford method or BCA kit (Thermo Fisher Scientific, Bartlett, IL, USA) was used to quantify protein concentrations (Bradford, 1976).

### Immunohistochemistry (IHC)

The left-brain hemisphere was immersion fixed in 4% paraformaldehyde, replaced with 30% w/v sucrose in PBS three times. Briefly, the brains were then embedded in 15% gelatin followed by immersion fixing in 4% paraformaldehyde, 15%, and 30% sucrose. The gelation blocks were serially sectioned (40 μm) *via* freezing microtome (Nagamoto-Combs et al., 2016). The serial sectioned brains were immunostained using anti-Aβ (4G8; 1:1,000 dilution), Iba-1 (1:1,000 dilution), GFAP (1:1,000 dilution), and CD68 (1:1,000 dilution), following citrate antigen retrieval, antibodies followed by their respective secondary antibodies. Vector VIP was used to visualize the immunoreactivity. Stained brain sections were scanned and imaged using a Hamamatsu 2.0 HT digital slide scanner. IHC quantitation was performed from a complete hippocampus section per mouse, totaling six mice per condition per each stain. The images were turned into grayscale first followed by inversion using Adobe Photoshop software (Adobe Systems, San Jose, CA, USA). The mean optical density per each image was recorded by selecting the whole hippocampus and averaged ± SEM.

### Western Blotting

Flash-frozen hippocampi and livers were lysed in RIPA buffer (20 mM Tris, pH 7.4, 150 mM NaCl, 1 mM Na_3_VO_4_, 10 mM NaF, 1 mM EDTA, 1 mM EGTA, 0.2 mM phenylmethylsulfonyl fluoride, 1% Triton X-100, 0.1% SDS, and 0.5% deoxycholate) with protease inhibitors (AEBSF 1 mM, Aprotinin 0.8 M, Leupeptin 21 M, Bestatin 36 M, Pepstatin A 15 M, E-64 14 M), centrifuged (14,000 rpm, 4°C, 10 min), and the supernatants were collected. Hippocampal proteins were resolved by 10% sodium dodecyl sulfate-polyacrylamide gel electrophoresis (SDS-PAGE) and transferred to polyvinylidene difluoride membranes (PVDF) for western blotting using anti- p-IR/IGF1-R, IGF-1R β, p-AKT, AKT, p-JNK, JNK, p-ERK, ERK, p-tyrosine, AβPP (Y188), CD68, GFAP, and α-tubulin (loading control) antibodies. For the liver western blotting, anti- p-IR/IGF1-R, IGF-1R β, and actin (loading control) antibodies were used. The BCA kit (Thermo Fisher Scientific, IL, USA) was used to quantify protein concentrations. Enhanced chemiluminescence was used to detect antibody binding using an Aplegen Omega Lum G imaging system. Optical density values were normalized to their respective loading controls (p-IGF-1R, p-AKT, p-JNK, and p-ERK blots against their respective IGF-1R, AKT, JNK, and ERK total proteins and p-tyrosine, AβPP, CD68, and GFAP blots against α-tubulin) from the same membrane, and then averaged (± SEM).

### Dot Blot

Total, oligomeric, and fibrillar protein from the hippocampal lysates in RIPA buffer were dot blotted onto PVDF membranes and detected using 6E10, A11, and OC antibodies, respectively. Enhanced chemiluminescence was used to detect antibody binding using an Aplegen Omega Lum G imaging system. Optical density values were normalized to α-tubulin, loading control.

### MTT Assay

Microglia were immediately used upon isolation by stimulating in serum-free DMEM/F12 with or without 100 ng/ml IGF-1, 25 ng/ml LPS from E coli O111:B4 L-4391 (Sigma-Aldrich, St. Louis, MO, USA), or 100 nM Aβ_42_ (A-1165, rPeptide, Athens, GA, USA) for 24 h. Aβ fibrils were resuspended with water at a 250 μM concentration for 5 min and slowly inverted to mix and allowed to incubate at 37°C for 96 h. Aβ conformation was confirmed *via* western blots for each experiment. MTT reagent [3-(4,5-dimethylthiazol-2-yl)-2,5-diphenyl tetrazolium bromide] was added to the media at a concentration of 100 μg/ml for 1 h. The media was removed and replaced with isopropanol. Optical density was read at 560 nm and 650 nm corrected wavelength on a Bio-Tek ELx800 plate reader (Winooski, VT, USA).

### Phagocytosis Assay

Microglia were immediately used upon isolation by stimulating in serum-free DMEM/F12 with or without 100 ng/ml IGF-1, 1 μM FITC-human Aβ_42_, or FITC-*Escherichia coli* bioparticle as a positive control, 0.125 mg/ml. Phagocytosis was measured by the uptake of fluorescein isothiocyanate (FITC)-conjugated Aβ_42_. The Aβ_42_ peptide was fibrillized as recommended by the manufacturer (rPeptide, Athens, GA, USA) and previously described (Floden and Combs, 2006). Cells were treated for 6 h, media was removed, and the wells were rinsed with 0.25 mg/ml trypan blue in PBS to quench any extracellular peptide or bioparticles that were not internalized. The intracellular fluorescence was read at 480 nm excitation and 520 nm emission *via* Bio-Tek FLx800 fluorescent plate reader (Winooski, VT, USA).

### Culture Media Enzyme-Linked Immunosorbent Assays (ELISA)

Microglia were immediately used upon isolation by stimulating in serum-free DMEM/F12 with or without 100 ng/ml IGF-1, 25 ng/ml LPS from E coli O111:B4 L-4391 (Sigma-Aldrich, St. Louis, MO, USA), or 100 nM Aβ_42_ (A-1165, rPeptide, Athens, GA, USA) for 24 h. Aβ fibrils were resuspended with water at a 250 μM concentration for 5 min and slowly inverted to mix and allowed to incubate at 37°C for 96 h. Aβ conformation was confirmed *via* western blots for each experiment. The media was removed and TNF-α levels quantified using a mouse TNF-α DuoSet ELISA kit according to the manufacturer’s protocol (R&D Systems, Minneapolis, MN, USA). The optical density was read on a Bio-Tek ELx800 plate reader (Winooski, VT, USA) at 450 nm and 550 nm corrected wavelength and pg/ml TNF-α was calculated *via* standard curve.

### Statistical Analysis

The *in vivo* data were analyzed by two-way ANOVA. The errors from comparisons of several pairs of means in the experiment were adjusted using Tukey’s multiple comparison tests using GraphPad Prism 8 software. One-way ANOVA followed by Dunnet’s *post hoc* test was used to analyze the *in vitro* data including the MTT assays, LDH assays, phagocytosis assays, and TNF-α ELISAs. Data are represented as the mean ± SEM. Significance is indicated by *P*-value measurements with a *P* < 0.05 considered significant; *P < 0.05.

## Results

### Aβ-Levels Were Attenuated by IGF-1R Inhibitor Treatment of AβPP/PS1 Mice

**Figure 1:**
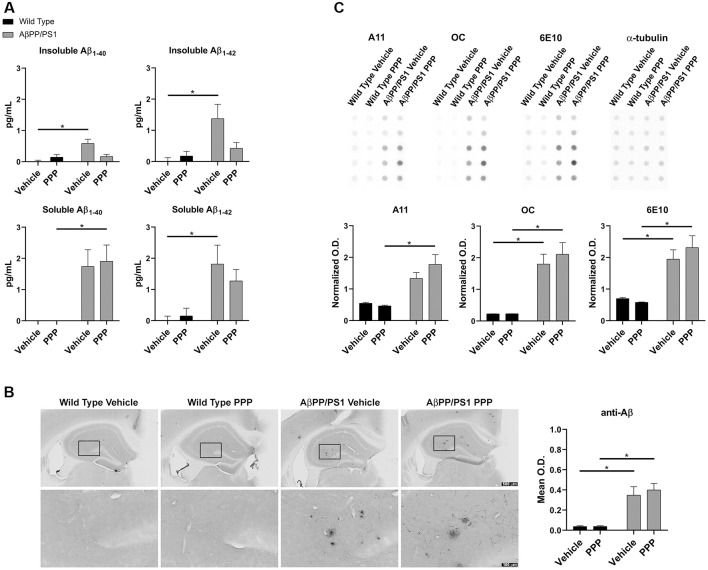
Insulin-like growth factor-1 receptor (IGF-1R) inhibitor treatment attenuated Aβ levels in AβPP/PS1 mice. **(A)** Temporal cortex lysates collected from male wild type (WT) and AβPP/PS1 mice, treated with vehicle (DMSO) or picropodophyllin (PPP, 1 mg/kg) for 7 days, were used to perform ELISAs for insoluble and soluble Aβ_1–40/42_. Data are mean ± SEM, *p < 0.05 (*n* = 7–9 animals). Two-way ANOVA indicate insoluble Aβ_1–40_ *F*_(1,24)_ = 9.576 and *p*(interaction) = 0.0050; soluble Aβ_1–40_ *F*_(1,23)_ = 0.4255 and *p*(interaction) = 0.5207; insoluble Aβ_1–42_ *F*_(1,26)_ = 4.896 and *p*(interaction) = 0.0359; soluble Aβ_1–42_ *F*_(1,24)_ = 0.8341 and *p*(interaction) = 0.3702. The errors from comparisons of several pairs of means in the experiment were adjusted using Tukey’s multiple comparison tests. **(B)** Immunohistochemistry (IHC) was performed on both WT and AβPP/PS1 mouse brains using anti-Aβ (4G8) antibody and the mean optical density of hippocampal staining was measured. Data are mean O.D. ± SEM, *p < 0.05 (*n* = 6 animals/condition). Two-way ANOVA indicate Aβ IHC *F*_(1,20)_ = 0.2204 and *p*(interaction) = 0.6438. The errors from comparisons of several pairs of means in the experiment were adjusted using Tukey’s multiple comparison tests. Representative hippocampal images are shown. **(C)** Hippocampal lysates from vehicle and PPP treated WT and AβPP/PS1 mice were dot blotted to quantify total Aβ (6E10), oligomeric peptide (A11), and fibrillar peptide (OC) changes. Data are mean O.D. ± SEM, *p < 0.05 (*n* = 5 animals). Two-way ANOVA indicate A11 dot blot *F*_(1,16)_ = 2.147 and *p*(interaction) = 0.1623; OC dot blot *F*_(1,16)_ = 0.4042 and *p*(interaction) = 0.5339; 6E10 dot blot *F*_(1,16)_ = 1.032 and *p*(interaction) = 0.3249. The errors from comparisons of several pairs of means in the experiment were adjusted using Tukey’s multiple comparison tests.

The inhibitory effect of the drug on IGF-1R phosphorylation was first confirmed *via* ip. injection of 1 mg/kg PPP and examining hippocampal lysates 15 min after the injection. Western blot results showed downregulation of p-IGF-1R and p-tyrosine levels (Supplementary Figure S1). Based upon this dosage, both WT and AβPP/PS1 lines (9–10-month-old) were treated with 1 mg/kg/day PPP to determine the impact of IGF-1R inhibition on Aβ deposition. Since the hippocampus and temporal cortex are areas of relevance in AD pathophysiology, Aβ changes were examined in both. The larger amount of tissue represented by the temporal cortex allowed this region to be used for ELISA analyses. ELISA indicated a decrease of insoluble Aβ_1–40_ and soluble/insoluble Aβ_1–42_ but not soluble Aβ_1–40_ levels in temporal cortices of AβPP/PS1 mice treated with PPP as levels were no longer significant from wild type controls (Figure 1A). Anti-Aβ IHC quantitation of hippocampal regions revealed no dramatic Aβ level changes (Figure 1B). However, plaque staining intensity appeared to be qualitatively reduced in the drug-treated AD mice vs. the vehicle-treated group, seen *via* higher magnification (Figure 1B). As an additional means of addressing changes in Aβ conformations, we performed dot blot analyses from hippocampal lysates using an anti-oligomer antibody, A11, anti-fibril antibody, OC, and total Aβ antibody, 6E10. Dot blots showed an increase in AβPP/PS1 A11 detectable protein following PPP treatment compared to PPP treated wild type controls (Figure 1C). Contrary to our expectations, the dot blot results did not show a decrease in OC immunodetection as was predicted from the ELISA, perhaps due to a lack of specificity of OC for Aβ_1–40/42_ only (Figure 1C). These findings suggest that Aβ deposition was affected by the IGF-1R function.

### Gliosis Immunoreactivity Was Not Altered in PPP Treated AβPP/PS1 Mice

**Figure 2:**
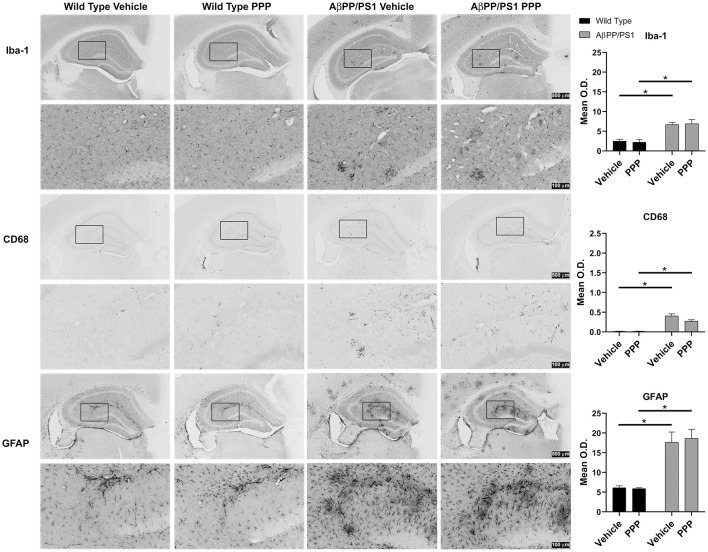
IGF-1R inhibitor treatment did not alter gliosis immunoreactivity in AβPP/PS1 mice. IHC was performed from the vehicle (DMSO) and PPP (1 mg/kg, 7 days) treated WT and AβPP/PS1 mouse brains using anti-Iba1 (microglial marker), anti-CD68 (microglia marker), and anti-GFAP (astrocyte marker) antibodies and mean optical density was measured. Antibody binding was visualized using Vector VIP as the chromogen. Data are mean O.D. ± SEM, *p < 0.05 (*n* = 6 animals/condition). Two-way ANOVA indicate Iba-1 staining *F*_(1,20)_ = 0.09309 and *p*(interaction) = 0.7634; CD68 staining *F*_(1,20)_ = 3.775 and *p*(interaction) = 0.0662; GFAP staining *F*_(1,20)_ = 0.1156 and *p*(interaction) = 0.7374. The errors from comparisons of several pairs of means in the experiment were adjusted using Tukey’s multiple comparison tests. Representative hippocampal images are shown.

Brain inflammatory changes are hypothesized to be a critical component of AD pathophysiology mediated, in part, by Aβ-stimulated microglial activation and expression of inflammatory cytokines in AD brains (Itagaki et al., 1989; Prinz et al., 2011; Choi et al., 2013; Wang et al., 2015). Based on the attenuated insoluble Aβ levels in the PPP treated AβPP/PS1 mice, we expected that inactivation of IGF-1R signaling *via* PPP would also alter reactive gliosis. To assess the impact of PPP treatment on glial activation, IHC was performed. Microglial activation was visualized using two different antibodies, anti-Iba-1 and anti-CD68. PPP treatment did not significantly alter the Aβ-associated hippocampal immunoreactivity for CD68 or Iba-1 antibody (Figure 2). Astrocyte activation, assessed using anti-GFAP immunoreactivity, also did not demonstrate any robust changes following PPP treatment (Figure 2).

### PPP Decreased Protein Markers of Microglial Activation and IGF-1R Signaling

**Figure 3:**
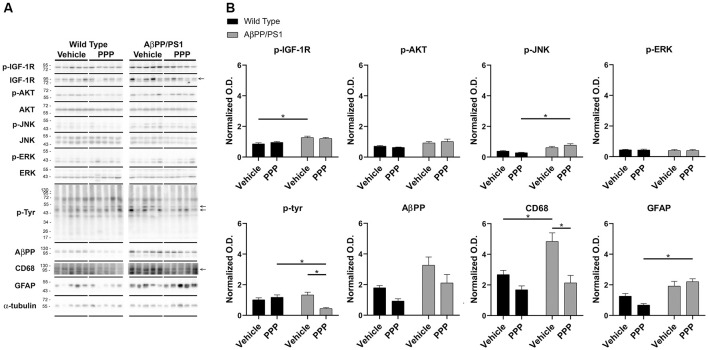
p-tyrosine and CD68 levels were attenuated by inhibitor treatment in AβPP/PS1 mice. Hippocampal lysates from vehicle and PPP (1 mg/kg, 7 days) treated WT and AβPP/PS1 mice were **(A)** western blotted to **(B)** quantify protein changes related to IGF-1R signaling, microgliosis, and AβPP levels. p-IGF-1R, p-AKT, p-JNK, and p-ERK blots were normalized to their respective IGF-1R, AKT, JNK, and ERK loading controls. The p-tyrosine, AβPP, CD68, and GFAP blots were normalized to their respective α-tubulin loading controls. Data are mean O.D. ± SEM, *p < 0.05 (*n* = 5 animals). Two-way ANOVA indicate p-IGF-1R *F*_(1,16)_ = 1.047 and *p*(interaction) = 0.3215; p-Akt *F*_(1,16)_ = 0.8088 and *p*(interaction) = 0.3818; p-JNK *F*_(1,16)_ = 4.291 and *p*(interaction) = 0.0548; p-ERK *F*_(1,16)_ = 0.008505 and *p*(interaction) = 0.9277; p-Tyr *F*_(1,15)_ = 13.98 and *p*(interaction) = 0.0020; AβPP *F*_(1,15)_ = 0.1227 and *p*(interaction) = 0.7310; CD68 *F*_(1,15)_ = 3.929 and *p*(interaction) = 0.0661; GFAP *F*_(1,16)_ = 4.429 and *p*(interaction) = 0.0515. The errors from comparisons of several pairs of means in the experiment were adjusted using Tukey’s multiple comparison tests.

To provide an additional quantitative assessment of changes in gliosis and IGF-1 receptor inhibition following PPP treatment, western blot analyses of hippocampi were performed. To quantify PPP-dependent changes in IGF-1R signaling, protein levels of active IGF-1R, p-IGF-1R, were quantified. p-IGF-1R protein levels were increased in vehicle-treated AβPP/PS1 mice compared to vehicle-treated wild type mice with no difference between the PPP groups suggesting a disease-associated increase in IGF-1R signaling and some attenuation due to drug treatment (Figure 3). Kinases associated with the IGF-1R response were also compared in treated vs. untreated mice. Active p-Akt and p-JNK levels were not altered by disease although PPP treatment of AβPP/PS1 mice increased p-JNK levels compared to PPP treated wild type controls (Figure 3). Levels of active, p-ERK also did not differ between strain and treatment (Figure 3). As an additional means of quantifying gliosis, protein levels of CD68 and p-tyrosine were examined to assess microgliosis. Total protein p-tyrosine levels were assessed as in our previous study which indicated a tyrosine kinase-dependent activation of microglia in response to Aβ stimulation (Dhawan et al., 2012). PPP treated AβPP/PS1 mice demonstrated a significant decrease in both CD68 and p-tyrosine-protein levels indicating a drug-dependent decrease in microgliosis (Figure 3). Protein levels of AβPP were not significantly altered by PPP treatment in either wild type or AβPP/PS1 mice (Figure 3). Interestingly, quantitation of GFAP protein levels, as a marker of astrogliosis, demonstrated a significant increase in PPP treated AβPP/PS1 mice compared to PPP treated wild type controls suggesting drug treatment may slightly increase astrogliosis (Figure 3).

### PPP Treatment Altered Phosphorylation Levels of Liver IGF-1R

**Figure 4:**
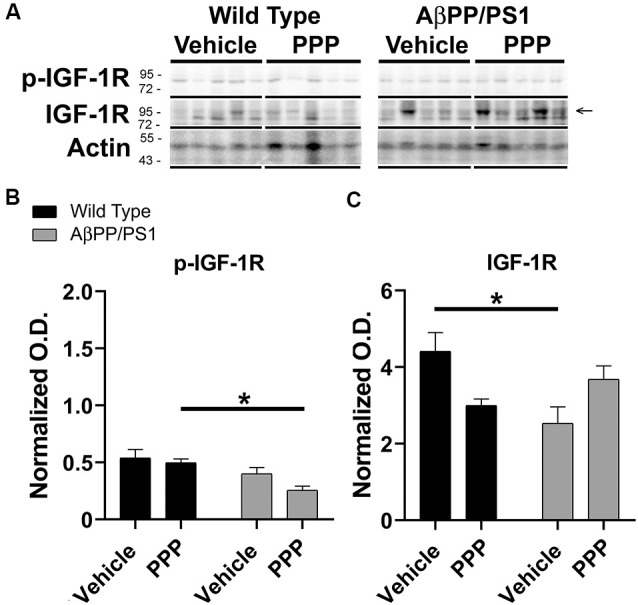
Liver p-IGF-1R levels were altered by inhibitor treatment in AβPP/PS1 mice. Liver lysates from the vehicle and PPP (1 mg/kg, 7 days) treated WT and AβPP/PS1 mice were **(A)** western blotted to quantify **(B)** p-IGF-1R and **(C)** IGF-1R protein changes. p-IGF-1R blots were normalized to their respective IGF-1R loading controls. Actin was used as a loading control for normalizing IGF-1R blots. Data are mean O.D. ± SEM, *p < 0.05 (*n* = 5 animals). Two-way ANOVA indicate p-IGF-1R *F*_(1,16)_ = 0.9666 and *p*(interaction) = 0.3402 and IGF-1R *F*_(1,16)_ = 11.39 and *p*(interaction) = 0.0039. The errors from comparisons of several pairs of means in the experiment were adjusted using Tukey’s multiple comparison tests.

Since the PPP-mediated inhibitory effect on microgliosis correlated with a reduction in brain levels of p-IGF-1R in AβPP/PS1 mice, we investigated whether the drug was exerting any maintained effect on peripheral p-IGF-1R levels. Based upon the high expression of IGF-1 and its receptor in the liver, we quantified liver phosphorylation levels of the receptor (Mauras, 1997). Unlike the brain, livers of AβPP/PS1 mice demonstrated no elevation in levels of p-IGF-1R compared to wild type mice demonstrating that the elevated activity associated with disease differs across organs and is perhaps unique to the brain (Figure 4). However, PPP treated AβPP/PS1 mice demonstrated a significant decrease in p-IGF-1R protein levels compared to the PPP treated wild type controls demonstrating drug effects on peripheral IGF-1R signaling in AβPP/PS1 mice (Figure 4). Surprisingly, vehicle-treated AβPP/PS1 mice had reduced total IGF-1R protein levels in the liver compared to vehicle-treated wild type controls (Figure 4). This suggests that peripheral, as well as brain IGF-1R function, may be altered in AβPP/PS1 mice.

### PPP Treatment Did Not Alter Cytokine Levels in the Temporal Cortex

**Figure 5:**
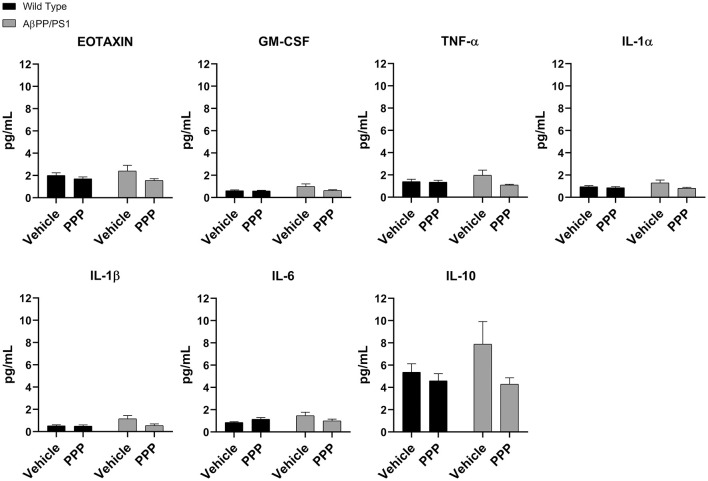
IGF-1R inhibitor treatment did not alter cytokines in the brains of AβPP/PS1 mice. ELISAs were performed to quantify levels of cytokines in temporal cortex lysates from WT and AβPP/PS1 mice that were vehicle or PPP (1 mg/kg, 7 days) treated. Data are mean values normalized to tissue wet weight ± SEM (*n* = 6–8 animals). Two-way ANOVA *p*(interaction) = 0.1805; Eotaxin *F*_(1,24)_ = 0.9376 and *p*(interaction) = 0.3426; GM-CSF *F*_(1,23)_ = 1.908 and *p*(interaction) = 0.1805; TNF-α *F*_(1,24)_ = 2.932 and *p*(interaction) = 0.0997; IL-1α *F*_(1,24)_ = 1.900 and *p*(interaction) = 0.1808; IL-1β *F*_(1,24)_ = 3.203 and *p*(interaction) = 0.0861; IL-6 *F*_(1,24)_ = 4.326 and *p*(interaction) = 0.0484; IL-10 *F*_(1,24)_ = 1.809 and *p*(interaction) = 0.1911. The errors from comparisons of several pairs of means in the experiment were adjusted using Tukey’s multiple comparison tests.

Due to the abundance of samples, temporal cortices rather than the hippocampus were used for ELISA analyses. Cytokine levels were quantified from the temporal cortices of vehicle and PPP treated mice to correlate with the observed changes in microglial activation. Surprisingly, PPP treatment did not affect cytokine levels across treatment or genotype groups when examining eotaxin, GM-CSF, TNF-α, IL-1α, IL-1β, IL-6, and IL-10 (Figure 5). The levels of other cytokines also did not change across strains or drug treatment (Supplementary Figure S2). These data indicate that the slight changes in markers of microgliosis and astrogliosis do not correlate with observable cytokine differences in the brain.

### Spleen Cytokine Levels Were Not Altered by PPP Treatment

To assess the effect of PPP treatment on peripheral inflammatory markers, spleen cytokine ELISAs were also performed. Vehicle treated AβPP/PS1 mice had no elevation of spleen cytokine levels compared to vehicle-treated wild type mice (Supplementary Figure S3). Also, PPP treatment did not alter the concentrations of any cytokines in either strain supporting the finding of a similar lack of change in the brain (Supplementary Figure S3).

### IGF-1R Stimulation Did Not Alter Microglial TNF-α Secretion but Modestly Affected Aβ Uptake *in vitro*

**Figure 6:**
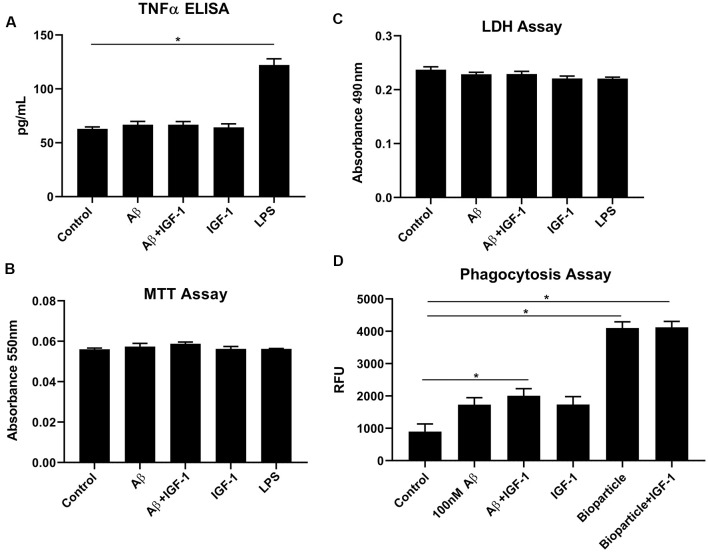
IGF-1 stimulation did not alter microglial cytokine secretory ability but modestly altered uptake in response to Aβ stimulation. Microglia were isolated from 10-month-old C57BL/6 wild type brains and stimulated in serum-free DMEM/F12 for 24 h with or without 100 nM Aβ_42,_ 25 ng/ml LPS or 100 ng/ml IGF-1. **(A)** TNF-α secretion was quantified *via* ELISA from the media, Graphs are means values ± SEM with replicates of 8, *p < 0.05. Representative data from three independent experiments are shown. One-way ANOVA indicates TNF-α *F*_(6,28)_ = 41.40 and *p* < 0.0001. Multiple comparisons were performed *via* Dunnet’s test. **(B)** Mitochondrial dehydrogenase activity in the cells was assessed *via* an MTT assay. Graphs are mean values ± SEM with replicates of 8. Representative data from three independent experiments are shown. One-way ANOVA indicates MTT *F*_(6,28)_ = 1.41 and *p* = 0.2458. Multiple comparisons were performed *via* Dunnet’s test. **(C)** Cell viability was quantified by an LDH release assay. Graphs are mean values ± SEM with replicates of 8. Representative data from three independent experiments are shown. One-way ANOVA indicate LDH *F*_(6,28)_ = 2.7 and *p* = 0.0339. Multiple comparisons were performed *via* Dunnet’s test. **(D)** 1 μM FITC-Aβ_42_ or 0.25 mg/ml FITC *E. coli* bioparticle positive control were added to the cells for 6 h with or without 100 ng/ml IGF-1 to assess phagocytic uptake. Graphs are mean values ± SEM with replicates of 8, *p < 0.05. Representative data from three independent experiments are shown. One-way ANOVA *F*_(5,35)_ = 39.33 and *p* < 0.0001. Multiple comparisons were performed *via* Dunnet’s test.

IGF-1R inhibitor significantly modified microglial phenotype and cytokine levels in AβPP/PS1 mouse brains. To test the idea that the effects of IGF-1R inhibition we observed were mediated by primarily altering microglial behavior, we examined the effect of IGF-1 stimulation on microglial phenotype *in vitro*. Microglia acutely isolated from adult wild type mouse brains were stimulated with or without IGF-1, Aβ_1–42_ fibrils as a disease-relevant ligand or LPS as a positive control to quantify changes in cytokine secretion, mitochondrial dehydrogenase activity, and LDH release. As, we have previously demonstrated, adult microglia did not respond to Aβ fibril stimulation by increasing TNF-α secretion (Figure 6; Floden and Combs, 2006). IGF-1 stimulation did not affect either basal TNF-α secretion or Aβ-stimulated TNF-α secretion (Figure 6). Similarly, IGF-1 in the absence or presence of Aβ did not affect mitochondrial dehydrogenase activity, as assessed by an MTT assay, or cell viability, as assessed by LDH release (Figure 6). On the other hand, incubation of the microglia with FITC-conjugated Aβ resulted in a slight increase in microglial uptake of peptide only in the presence of concomitant IGF-1 stimulation (Figure 6). IGF-1 stimulation could not alter the microglial uptake of bacterial Bioparticle (Figure 6). This data suggested that IGF-1 has a minimal ability to alter microglial phenotype providing support for the idea that elevated IGF-1 in the AβPP/PS1 brains may be exerting effects on microglia.

## Discussion

We observed that reversible pharmacologic inhibition of IGF-1R *via* PPP reduced insoluble Aβ_1–40_ and soluble/insoluble Aβ_1–42_ levels in temporal cortices of 9–10-month-old AβPP/PS1 mice. Although the histologic analysis was unremarkable, western blot findings demonstrated that PPP treatment attenuated brain p-IGF-1R levels in AβPP/PS1 mice correlating with decreased protein levels of p-tyrosine and CD68 as markers of reactive microglia and a slight increase in astrocytic activation assessed by GFAP western blot. Despite the changes in Aβ levels and protein markers of gliosis, no corresponding changes in brain cytokines were observed.

Previously, we demonstrated higher levels of IGF-1 in the parietal cortex but not the hippocampus of AβPP/PS1 mice at 6 months of age compared to WT mice (Puig et al., 2016). The present data also revealed no significant differences in IGF-1 levels between these two genotypes in the temporal cortices of 9–10-month-old mice before or after the drug exposure (Supplementary Figure S2). Consistent with our prior work, this suggests that there is the heterogeneity of IGF-1 levels in brain regions. Besides local expression of IGF-1, roughly 95% of this protein is transported from the liver to the brain crossing the blood-brain barrier suggesting there may be some regional selectivity of import (Yamamoto and Murphy, 1995; Carro et al., 2000). Also, higher levels of serum IGF-1 have been demonstrated both in 3xTg-AD mice and AD patients compared to controls (Vardy et al., 2007; Parrella et al., 2013). Thus, to investigate the levels of local brain IGF-1, the circulating IGF-1, and its transport into the brain must be considered in future work (Adams et al., 2009). Nevertheless, our data demonstrate that transient pharmacologic inhibition of the brain IGF-1R is a feasible strategy, in addition to the genetic elimination of IGF-1 or its receptor, to study the effect of this growth factor in the CNS.

Much of the mechanistic effects of IGF-1R signaling inhibition related to AD using inhibitors or genetic deletion of the receptor rely on *in vitro* studies. For example, like IGF-1, Aβ_1–42_ monomers reportedly activate the IGF-1R/PI3K/Akt/CREB pathway in mature cultures of pure cortical neurons after a 30 min exposure. CREB (cyclic adenosine monophosphate response element-binding protein) activation induces expression of BDNF (brain-derived neurotrophic factor) involved in memory formation. PPP and LY294002, the PI3K inhibitor, reverse this effect (Zimbone et al., 2018). Similarly, activated IGF-1R, due to Aβ_1–42_ monomer stimulation, promotes glucose uptake in cultured neurons at physiological concentrations. This is also inhibited by PPP treatment (Giuffrida et al., 2015). In contrast, Aβ_1–42_ monomers, under pathological conditions, accumulate into oligomers leading to neuronal and synaptic loss (Giuffrida et al., 2015). Even though Aβ oligomers show attenuating effects on active p-CREB levels, no changes were observed on p-Akt levels *in vitro* (Zimbone et al., 2018) indicating direct activation of the IGF-1R pathway *via* monomeric Aβ_1–42_. This suggests that Aβ oligomers may not downregulate p-CREB *via* inhibiting the IGF-1R/Akt signaling pathway since CREB is also activated by other signaling pathways (Ortega-Martínez, 2015). In another study, astrocyte-specific IGF-1R knockout cultures demonstrated increased mitochondrial ROS production and diminished Aβ_1–42_ uptake (Logan et al., 2018). These studies suggest distinct roles of IGF-1R in different cell types that support both potentiating and attenuating functions during disease.

Human and AD mouse model studies also demonstrate the dichotomous effects of IGF-1R in AD. Similar to our data, several human and animal AD studies support an exacerbating role for IGF-1R signaling in AD. For instance, hyperactivation of the PI3K/Akt/mTOR pathway has been reported in post-mortem tissue from the inferior parietal lobe of amnestic mild cognitive impairment and AD patients compared to controls. Activation of this pathway leads to protein synthesis/protein homeostasis and downregulation of autophagy/protein clearance (Tramutola et al., 2015). A decrease in autophagy function, as a result of PI3K/Akt/mTOR pathway activation, is associated with the aggregation of Aβ_1–42_ proteins in the brain (Tramutola et al., 2015). Similarly, Aβ accumulation upregulates mTOR signaling and pharmacological inhibition of mTOR with rapamycin decreases Aβ_1–42_ and tau levels and alleviates cognitive deficits by promoting autophagy in an AD mouse model (Caccamo et al., 2010). In another study, a natural plant extract, curcumin, led to induction of autophagy, inhibition of Aβ production, and cognitive improvement *via* attenuating the PI3K/Akt/mTOR pathway in AβPP/PS1 mice (Wang et al., 2014). Additionally, a beneficial effect of inhibiting the insulin/IGF-1 signaling cascade *via* NT219 has been reported in nematodes. The inhibitor-induced a reduction of p-IGF-1R and degradation of the IR substrates 1 and 2 (IRS1/2) leading to the enhanced stress resistance and protection against Aβ and polyQ_40_ proteotoxicity, AD and Huntington’s disease-associated proteins, respectively (El-Ami et al., 2014). However, others have reported beneficial effects of IGF-1R signaling in AD. A sporadic AD rat model which was developed by intracerebral administration of streptozotocin (STZ) revealed increased AD characteristics including, p-tau, Aβ_42_, and neuroinflammation, and impaired IGF-1R/Akt signaling. However, T3D-959 treatment, an orally active brain-penetrating PPARδ/γ dual nuclear receptor agonist and a potent insulin sensitizer, ameliorated cognitive deficits and AD pathological hallmarks *via* promoted expression of insulin/IGF-1/Akt signaling proteins in the temporal lobes (de la Monte et al., 2017). Except for the latter study, many investigations of IGF-1R signaling, including our own, were performed using AβPP/PS1 mice which is a familial AD model that overexpresses mutant AβPP/PS1. The AβPP/PS1 model demonstrates robust plaque deposits but no p-tau containing neurofibrillary tangles as a limitation that may ultimately alter our ability to extrapolate findings to human disease. Collectively, these data illustrate the complexity of IGF-1 and its associated receptor in mediating changes in the transgenic mice and suggest that therapeutic strategies that interfere with IGF-1R signaling should consider age, transgenic mouse model, disease state, and even the confirmation of Aβ.

It was notable that IGF-1R inhibition demonstrated attenuating effects for insoluble Aβ_1–40_ and soluble/insoluble Aβ_1–42_ but not soluble Aβ_1–40_ in our study (Figure 1). This is partially consistent with results from Gontier et al. (2015) who generated an ADINKO model *via* knocking out the IGF-1R gene in adult neurons of AβPP/PS1 mice by injecting tamoxifen at 2 months of age. They reported decreased Aβ monomer, oligomer, insoluble Aβ, and amyloid plaque density correlating with the reduction of both proteotoxicity and neuronal loss in the forebrain (Gontier et al., 2015). Interestingly, similar results, including decreased Aβ pathology, were not observed by ablating the neuronal IGF-1R in 17 month-old AβPP/PS1 (ADINKO) mice when they had already developed advanced disease symptoms (George et al., 2017). Curiously, our drug treatment with the IGF-1R inhibitor decreased insoluble but not soluble levels of Aβ_1–40_. One possible explanation for this difference in Aβ conformation changes is that fibril clearance or aggregation is altered by inhibiting IGF-1R. Collectively, these data illustrate the complexity of IGF-1 and its associated receptor in mediating changes in the transgenic mice and suggest that anti-amyloid therapeutic strategies that interfere with IGF-1R signaling may be dependent upon age, particular cell type, disease state, and even the conformation of Aβ.

Despite changes in gliosis and Aβ levels, we observed no alteration of brain cytokine levels due to PPP treatment (Figure 5). This is in contrast to prior work demonstrating immune regulatory consequences of IGF-1R inhibition. Others have demonstrated that 16 months of rapamycin administration decreases hippocampal IL-1β levels correlating with improved learning and memory (Majumder et al., 2012). Also, intracerebroventricular injection of Aβ oligomers, which induces synaptic and cognitive deficits, failed to induce neuroinflammation and behavioral dysfunction in neuronal IGF-1R knockout mice (Lesné et al., 2013; Clarke et al., 2015; Ferreira et al., 2015; George et al., 2017). One possibility for our lack of change in brain cytokines may be that a longer PPP treatment time or increased dosage may be required to observe differences. Alternatively, analysis of hippocampal cytokine changes might have reflected brain region-specific effects.

Our strategy for assessing beneficial effects of attenuated IGF-1R signaling relied on the use of PPP, a reversible, selective inhibitor of the IGF-1R which preferentially targets the PI3K/Akt pathway (Vasilcanu et al., 2004). Also, this drug is reported to be brain penetrant although our current study did not quantify this (Yin et al., 2010). PPP treatment is expected to inhibit the phosphorylation of IGF-1R, but no other tyrosine kinase receptors (Girnita et al., 2004; Vasilcanu et al., 2008). PI3K/Akt/mTOR is considered a primary signaling response of the IGF-1R (Gontier et al., 2015) and therefore a likely target for modulating and assessing IGF-1R effects in the brain. Indeed, elevated mTOR signaling has been reported in the 3xTg AD mice at 6 and 12 months of age as well as in AD brains (Pei and Hugon, 2008; Caccamo et al., 2010; Oddo, 2012; Tramutola et al., 2015). Consistent with this, higher levels of phosphorylated Akt in 6-month-old AβPP/PS1 mice compared to controls have also been reported. However, 17 month-old AβPP/PS1 mice demonstrate lower levels of Akt phosphorylation compared to their respective controls (George et al., 2017). Similarly, we also observed increased levels of phosphorylated IGF-1R, but not Akt, in 9–10-month-old AβPP/PS1 mice compared to the WT controls (Figure 3). Collectively, these results suggest that IGF-1R signaling responses may vary depending upon the age of the mice or the transgenic mouse line. Also, the level of signaling response changes may be downstream of receptor phosphorylation itself.

Correlating with the clear brain effects of PPP administration we observed a slight change in levels of IGF-1R phosphorylation in the drug-treated hippocampi *via* western blot analysis (Figure 3). The only modest change observed is expected based upon the short half-life of PPP (2–4 h) and the fact that we collected mouse brains 1 day after the last injection of the inhibitor (Economou et al., 2008). Nevertheless, this encouraging effect provided some assurance that IGF-1R inhibition can be targeted in the brain. Our global assessment of tyrosine kinase-based signaling responses *via* anti-p-tyrosine western blotting also demonstrated a significant decrease in protein levels in the drug-treated mouse brains suggesting that some persistent signaling responses were still decreased even 1 day after drug treatment that will need to be identified (Figure 3). Co-microinjection of PPP resulted in an attenuated increase of p75 neurotrophin receptor levels 24 h after injection in a prior work consistent with our findings of drug-dependent attenuation of protein changes (Ito et al., 2012). Possibly the drug treatment-dependent effects on acute IGF-1R-related signaling responses are transiently detectable as prior work has shown that rapamycin treatment did not change the levels of total or phosphorylated mTOR although it did decrease levels of Aβ_42_ and deposition, tau pathology, and early learning and memory impairments in 3xTg AD mice (Caccamo et al., 2010). By contrast, neuronal knock-out IGF-1R AD (ADINKO) mice demonstrated decreased cortical phosphorylated Akt/Akt (Gontier et al., 2015). This difference might be due to the long-term and irreversible inactivation of the IGF-1R response in that particular mouse model. Collectively, these findings support the idea that although pharmacologic manipulation of the IGF-1R signaling response results in significant changes in the brain, assessment of receptor signaling responses likely depends upon treatment times, and the analysis method. We employed a short-term treatment of 7 days with a minimal dosage of 1 mg/kg to exert reversible effects on IGF-1R signaling responsible for beneficial effects on Aβ levels and gliosis in these AD mice. Our pilot screening indicated the efficacy of the 1 mg/kg dosage for attenuating p-IGF-1R and p-tyrosine levels when brains were examined 15 min after drug administration (Supplementary Figure S1) that was maintained at least up to 24 h (Figure 3). This concentration is far less than prior work which used 10–20 mg/kg in mice to exert brain effects on central control of body temperature or glioma growth and produced no lasting effects on brain p-IGF-1R levels (Yin et al., 2010; Cintron-Colon et al., 2017).

We observed a somewhat cell-selective effect of attenuated microgliosis while astrogliosis was slightly increased in the hippocampus of the male AβPP/PS1 drug-treated group (Figures 2, 3). Interestingly, others have found similar results of attenuated microglial accumulation in the cortex and hippocampus of both genders of ADINKO mice (Gontier et al., 2015). Moreover, higher astrogliosis was reported in the hippocampus of female ADINKO mice without any changes in the female cortex or male ADINKO hippocampus and cortex vs. controls (Gontier et al., 2015). These results suggest that genetic, as well as pharmacological inactivation of IGF-1R, correlates with attenuated microgliosis with a linkage to associated changes in astrogliosis in different AD mouse models.

Perhaps this is not surprising given the fact that microglial changes including altered phagocytosis and cytokine secretion are associated with the progression of AD (McQuade and Blurton-Jones, 2019). According to our data, PPP administration attenuated microgliosis and total p-tyrosine levels in correlation with our previous report (Dhawan et al., 2012). This suggests that the immunomodulatory effects of IGF-1R inhibition in the brain may be mediated through altered microglial phenotype (Figure 3). Surprisingly, however, we observed a modest effect of IGF-1 stimulation on increasing microglial phagocytic ability for Aβ but not bacterial bioparticles indicating that stimulation had some selectivity for altering particular types of microglial uptake (Figure 6). However, IGF-1 stimulation did not alter basal or Aβ-stimulated TNF-α secretion from microglia although additional cytokines were not assessed (Figure 6). Our data indicate that IGF-1 stimulation of Aβ plaque-associated microglia *in vivo* may indeed be contributing to microglial phenotype changes.

Our *in vivo* study suggests that short-term pharmacologic inhibition of the IGF-1R can alter AD-related pathological features including Aβ levels and gliosis. Importantly, this effect appears to be different for microglia compared to astrocytes and insoluble Aβ compared to soluble. However, since the IGF-1R is key to biological pathways involved in the aging process and age-related diseases, additional work is needed to investigate the effect of chronic administration of PPP or other pharmacologic IGF-1R inhibitors on the progression of AD. Furthermore, a more comprehensive assessment of disease phenotype including behavioral outcomes corresponding with a complete evaluation of pharmacological suppression of IGF-1R signaling will be needed to better validate the IGF-1R as a valid long-term target for disease attenuation.

## Data Availability Statement

All data associated with this study are included in the article.

## Ethics Statement

The use of the animals and experimental procedures were reviewed and approved by the University of North Dakota (UND) Institutional Animal Care and Use Committee.

## Author Contributions

MS performed western blots, ELISA, immunohistochemistry, analyzed the data, drafted and edited the final manuscript. GM designed and performed animal experiments, ELISAs, analyzed the data, and edited the final manuscript. AF performed microglial experiments, analyzed the data, and edited the final manuscript. MK assisted with statistical analysis and reviewed the final manuscript. CC designed and performed animal experiments and edited the final version of the manuscript. All authors read and approved the final manuscript.

## Conflict of Interest

The authors declare that the research was conducted in the absence of any commercial or financial relationships that could be construed as a potential conflict of interest.

## Supplementary Material

FIGURE S1 Administration of PPP downregulated p-IGF-1R and p-tyrosine protein levels in AβPP/PS1 mice. Hippocampi from vehicle-treated wild type and AβPP/PS1, as well as PPP, treated AβPP/PS1 mice were collected 15 min following vehicle (DMSO) or 1 mg/kg PPP ip injection. Western blots were performed to investigate changes in protein levels including IGF-1R, p-IGF-1R, and p-tyrosine. GAPDH was used as the loading control.

FIGURE S2 PPP treatment did not affect the levels of cytokines in mouse temporal cortices. ELISAs were performed to quantify levels of cytokines in temporal cortex lysates from WT and AβPP/PS1 mice that were vehicle or PPP (1 mg/kg, 7 days) treated. Data are mean values normalized to tissue wet weight ± SEM (*n* = 6–8 animals). Two-way ANOVA indicate MCP-1 *F*_(1,24)_ = 1.407 and *p*(interaction) = 0.2471; IGF-1 *F*_(1,23)_ = 0.03572 and *p*(interaction) = 0.8517; IFN-γ *F*_(1,23)_ = 0.6121 and *p*(interaction) = 0.4420; IL-2 *F*_(1,24)_ = 0.9921 and *p*(interaction) = 0.3292; IL-4 *F*_(1,23)_ = 0.2221 and *p*(interaction) = 0.6419; IL-12 *F*_(1,24)_ = 0.4948 and *p*(interaction) = 0.4885; IL-17 *F*_(1,24)_ = 0.5381 and *p*(interaction) = 0.4703. The errors from comparisons of several pairs of means in the experiment were adjusted using Tukey’s multiple comparison tests.

FIGURE S3 IGF-1R inhibitor treatment exerted no effect on cytokine levels in the spleens of AβPP/PS1 mice. ELISAs were performed to quantify levels of cytokines in spleen lysates from the vehicle or PPP (1 mg/kg, 7 days) treated WT and AβPP/PS1 mice. Data are mean values normalized to tissue wet weight ± SEM (*n* = 5–7 animals). Two-way ANOVA indicate GM-CSF *F*_(1,20)_ = 0.0006634 and *p*(interaction) = 0.9797; MCP-1 *F*_(1,20)_ = 0.4284 and *p*(interaction) = 0.5202; IFN-γ *F*_(1,20)_ = 0.01967 and *p*(interaction) = 0.8899; TNF-α *F*_(1,20)_ = 0.003344 and *p*(interaction) = 0.9545; IL-1α *F*_(1,20)_ = 0.04753 and *p*(interaction) = 0.8296; IL-1β *F*_(1,20)_ = 0.1536 and *p*(interaction) = 0.6992; IL-2 *F*_(1,20)_ = 0.3636 and *p*(interaction) = 0.5533; IL-4 *F*_(1,20)_ = 0.004686 and *p*(interaction) = 0.9461; IL-6 *F*_(1,20)_ = 0.01765 and *p*(interaction) = 0.8956; IL-10 *F*_(1,20)_ = 0.03820 and *p*(interaction) = 0.8470; IL-12 *F*_(1,20)_ = 0.03639 and *p*(interaction) = 0.8506; IL-17 *F*_(1,20)_ = 0.1150 and *p*(interaction) = 0.7380. The errors from comparisons of several pairs of means in the experiment were adjusted using Tukey’s multiple comparison tests.

## Abbreviations

AD: Alzheimer’s disease
Aβ: Amyloid β
GH: Growth hormone
IGF-1: Insulin-like growth factor
IGF-1R: Insulin-like growth factor receptor
PPP: Picropodophyllin
IR: Insulin receptor.
