# Supplementary figures and images for: IGF-1R Inhibitor Ameliorates Neuroinflammation in an Alzheimer’s Disease Transgenic Mouse Model
Source: Front Cell Neurosci. 2020 Jul 3;14:200. doi: 10.3389/fncel.2020.00200 (PMC7348663; doi:10.3389/fncel.2020.00200)

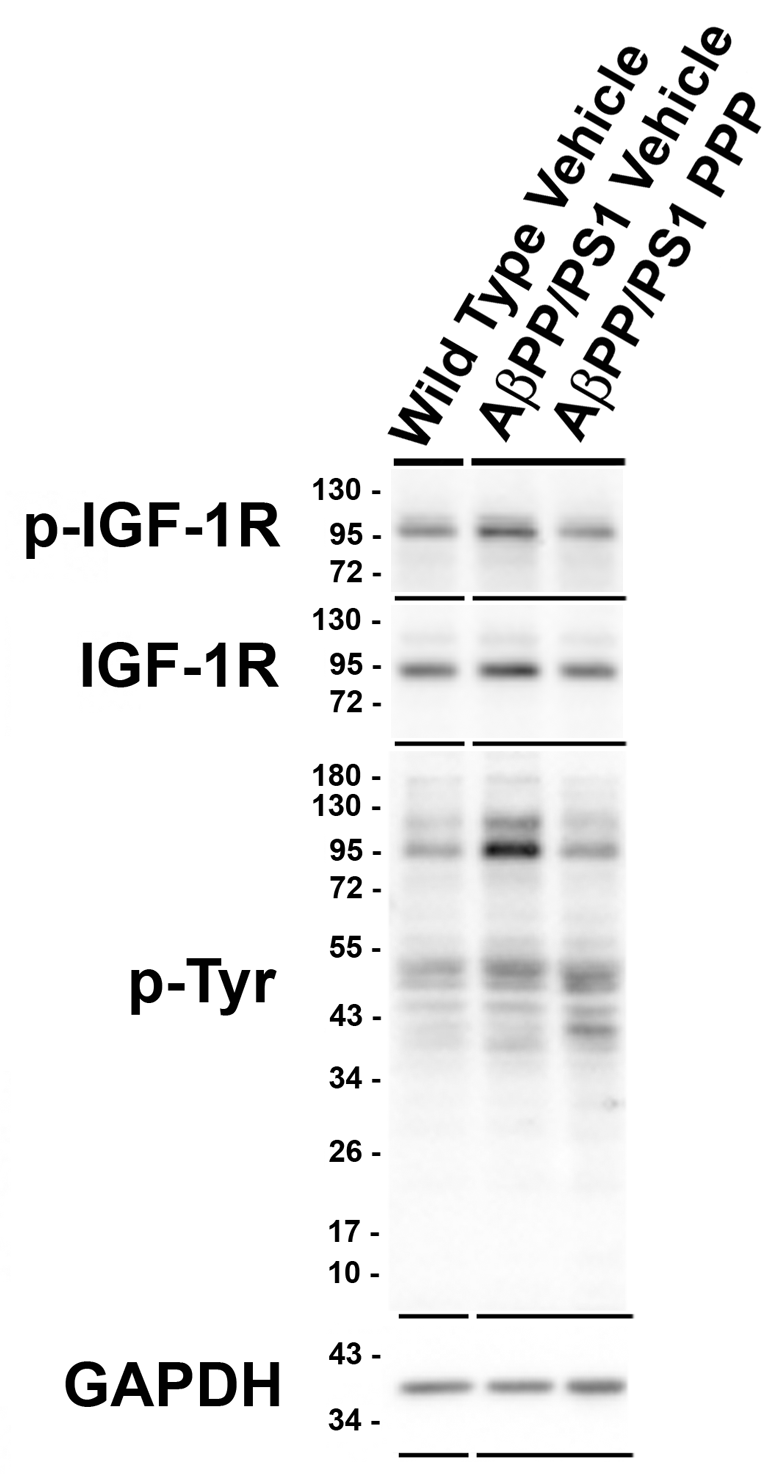

Supplement: FIGURE S1 — Administration of PPP downregulated p-IGF-1R and p-tyrosine protein levels in AβPP/PS1 mice. Hippocampi from vehicle-treated wild type and AβPP/PS1, as well as PPP, treated AβPP/PS1 mice were collected 15 min following vehicle (DMSO) or 1 mg/kg PPP ip injection. Western blots were performed to investigate changes in protein levels including IGF-1R, p-IGF-1R, and p-tyrosine. GAPDH was used as the loading control. [file Image_1.TIF]

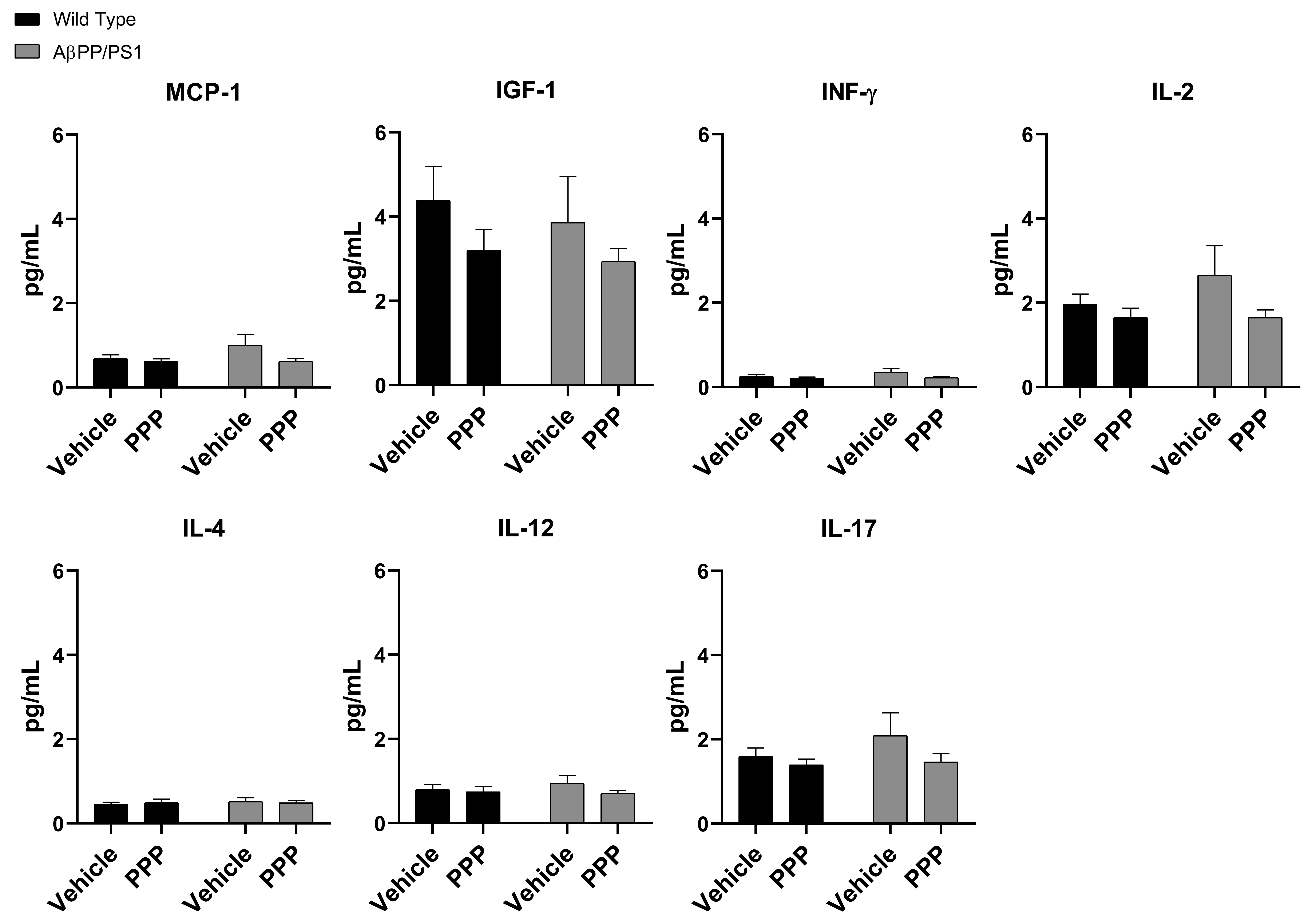

Supplement: FIGURE S2 — PPP treatment did not affect the levels of cytokines in mouse temporal cortices. ELISAs were performed to quantify levels of cytokines in temporal cortex lysates from WT and AβPP/PS1 mice that were vehicle or PPP (1 mg/kg, 7 days) treated. Data are mean values normalized to tissue wet weight ± SEM (n = 6–8 animals). Two-way ANOVA indicate MCP-1 F(1,24) = 1.407 and p(interaction) = 0.2471; IGF-1 F(1,23) = 0.03572 and p(interaction) = 0.8517; IFN-γ F(1,23) = 0.6121 and p(interaction) = 0.4420; IL-2 F(1,24) = 0.9921 and p(interaction) = 0.3292; IL-4 F(1,23) = 0.2221 and p(interaction) = 0.6419; IL-12 F(1,24) = 0.4948 and p(interaction) = 0.4885; IL-17 F(1,24) = 0.5381 and p(interaction) = 0.4703. The errors from comparisons of several pairs of means in the experiment were adjusted using Tukey’s multiple comparison tests. [file Image_2.TIF]

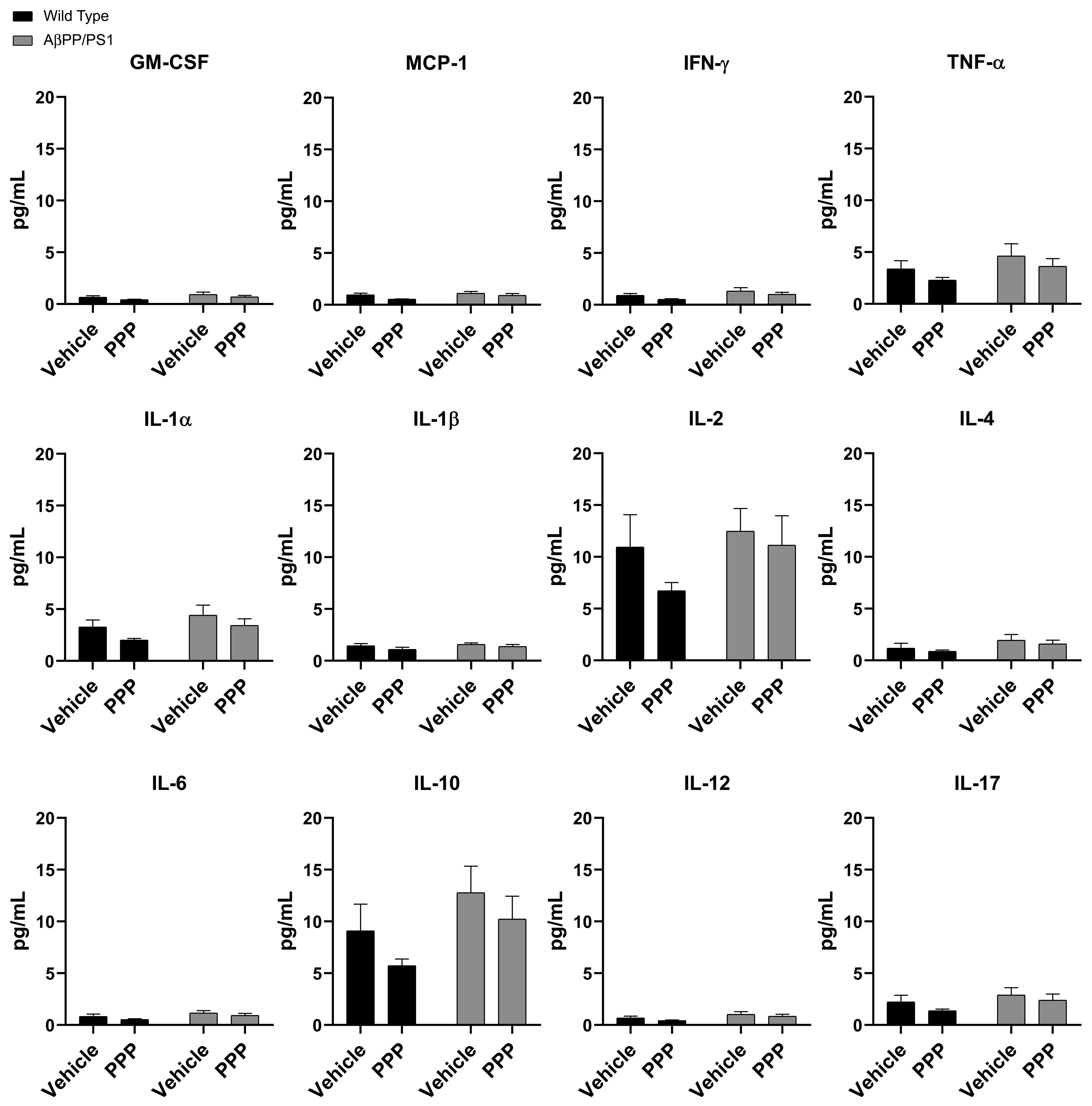

Supplement: FIGURE S3 — IGF-1R inhibitor treatment exerted no effect on cytokine levels in the spleens of AβPP/PS1 mice. ELISAs were performed to quantify levels of cytokines in spleen lysates from the vehicle or PPP (1 mg/kg, 7 days) treated WT and AβPP/PS1 mice. Data are mean values normalized to tissue wet weight ± SEM (n = 5–7 animals). Two-way ANOVA indicate GM-CSF F(1,20) = 0.0006634 and p(interaction) = 0.9797; MCP-1 F(1,20) = 0.4284 and p(interaction) = 0.5202; IFN-γ F(1,20) = 0.01967 and p(interaction) = 0.8899; TNF-α F(1,20) = 0.003344 and p(interaction) = 0.9545; IL-1α F(1,20) = 0.04753 and p(interaction) = 0.8296; IL-1β F(1,20) = 0.1536 and p(interaction) = 0.6992; IL-2 F(1,20) = 0.3636 and p(interaction) = 0.5533; IL-4 F(1,20) = 0.004686 and p(interaction) = 0.9461; IL-6 F(1,20) = 0.01765 and p(interaction) = 0.8956; IL-10 F(1,20) = 0.03820 and p(interaction) = 0.8470; IL-12 F(1,20) = 0.03639 and p(interaction) = 0.8506; IL-17 F(1,20) = 0.1150 and p(interaction) = 0.7380. The errors from comparisons of several pairs of means in the experiment were adjusted using Tukey’s multiple comparison tests. [file Image_3.TIF]
